# Integrating Tobacco Use Assessment and Treatment in the Oncology Setting: Quality Improvement Results from the Georgetown Lombardi Smoking Treatment and Recovery Program

**DOI:** 10.3390/curroncol30040285

**Published:** 2023-03-28

**Authors:** Kathryn L. Taylor, Marguerite A. Webster, Joanna G. Philips, Julia M. Whealan, Tania Lobo, Kimberly M. Davis, Chavalia J. Breece, Jennifer R. Wheeley, Jack E. Childs, Ariel Q. Le, Randi M. Williams, Irina G. Veytsman, Chul Kim

**Affiliations:** 1 Cancer Prevention and Control Program, Lombardi Comprehensive Cancer Center, Georgetown University Medical Center, Washington, DC 20007, USA; 2 Department of Psychiatry, Georgetown University Hospital, MedStar Health, Washington, DC 20007, USA; 3 Department of Pulmonary Medicine, MedStar Georgetown University Hospital, Washington, DC 20007, USA; 4 Department of Medical Oncology, MedStar Georgetown University Hospital, Washington, DC 20007, USA; 5 Department of Medical Oncology, MedStar Washington Hospital Center, Washington, DC 20010, USA; 6 Department of Medicine, Georgetown University Medical Center, Division of Hematology and Oncology, Lombardi Comprehensive Cancer Center, Washington, DC 20007, USA

**Keywords:** tobacco use assessment, tobacco use treatment, oncology patients, quality improvement, implementation strategies

## Abstract

As part of the NCI’s Cancer Center Cessation (C3i) initiative, we initiated, expanded, and maintained an evidence-based tobacco treatment program at the Georgetown Lombardi Comprehensive Cancer Center. We present a quality improvement (QI) assessment of the implementation process and patient-level outcomes. At two hematology/oncology outpatient clinical sites, five oncology-based teams (clinical administrators, clinical staff, pharmacy, information technology, and tobacco treatment staff) developed implementation strategies for opt-out patient assessment and enrollment, centralized tobacco treatment, audit, feedback, and staff training. Among eligible patients (tobacco use in ≤30 days), we assessed demographic, clinical, and tobacco-related characteristics to examine predictors of enrollment (baseline completed), treatment engagement (≥one sessions completed), and self-reported 7-day abstinence (6 months post-enrollment). Across both sites, medical assistants screened 19,344 (82.4%) patients for tobacco use, which identified 1345 (7.0%) current tobacco users, in addition to 213 clinician referrals. Of the 687/1256 (54.7%) eligible patients reached, 301 (43.8%) enrolled, and 199 (29.0%) engaged in treatment, of whom 74.5% were African American and 68% were female. At the larger site, significant multivariate predictors of enrollment included African American race (vs. white/other) and clinician referral (vs. MA assessment). Treatment engagement was predicted by greater nicotine dependence, and abstinence (27.4%) was predicted by greater treatment engagement. In summary, the systematic utilization of multiple oncology-based teams and implementation strategies resulted in the development and maintenance of a high-quality, population-based approach to tobacco treatment. Importantly, these strategies addressed inequities in tobacco treatment, as the program reached and engaged a majority-African-American patient population. Finally, the opt-out patient assessment strategy has been implemented in multiple oncology settings at MedStar Health through the Commission on Cancer’s Just Ask program.

## 1. Introduction

Among patients diagnosed with cancer who smoke, an estimated 20–30% continue smoking following diagnosis [1,2]. Continued tobacco use during and following cancer treatment contributes to poorer treatment outcomes, including increased risk of cancer-specific and all-cause mortality, cancer recurrence, second primary cancers, and treatment-related toxicities [3]. In contrast, smoking cessation can significantly improve health outcomes for cancer survivors [3,4]. Guidelines [5,6] recommend treating tobacco dependence as a routine part of cancer care. However, the implementation of oncology-based tobacco cessation programs has been limited until recently [7].

Between 2017 and 2020, the National Cancer Institute began the Cancer Center Cessation Initiative (C3i) to support the implementation of tobacco treatment programs at 52 NCI-designated cancer centers. The goal of C3i is to ensure that all cancer patients who smoke have access to smoking cessation support during and following treatment [7]. With C3i funding, we established the Smoking Treatment and Recovery (STAR) Program to provide evidence-based tobacco treatment to hematology and oncology patients at the Georgetown Lombardi Comprehensive Cancer Center, in collaboration with its clinical partner, MedStar Georgetown University Hospital (MGUH) [8]. In 2020, supported by MedStar Health, we implemented the STAR program at the Washington Cancer Institute of the MedStar Washington Hospital Center (MWHC), a regional cancer center that serves a diverse patient population, including racial and ethnic minority groups and patients with fewer socioeconomic resources. 

Several C3i programs have evaluated the barriers and facilitators of the successful implementation of smoking cessation [9,10,11,12]. One notable barrier has been the lack of involvement of information technology (IT) leadership, which is critical for completing the electronic health record (EHR) modifications needed to improve the documentation of tobacco use [13]. Additionally, patients’ lack of awareness of the harms of continued smoking after a cancer diagnosis is a barrier to engaging in smoking cessation treatment [14]. Facilitators of implementing tobacco treatment programs include the involvement of cancer center and healthcare system leadership (e.g., oncology and pharmacy administrators) [12]. Providing free nicotine replacement therapy (NRT) can facilitate patients’ engagement with treatment [15]. The objective of this paper is to describe the quality improvement assessment of the three primary strategies we utilized to facilitate the identification of eligible patients as well as treatment enrollment and engagement, and to present the patient outcomes that resulted following the use of these strategies [16].

### 1.1. Implementation Strategies

#### 1.1.1. Opt-Out Identification of Eligible Patients

Identifying patients who may benefit from tobacco treatment using a proactive, “opt-out” approach requires the assessment of all patients in a given population [17,18]. In traditional clinical care models that employ an “opt-in” approach, patients either accept or decline a clinician’s referral. This method typically limits tobacco treatment referrals to those who indicate that they are ready to quit, while excluding those who are not ready to quit but who may be willing to receive smoking cessation support [19]. Opt-out approaches can simultaneously reduce clinician burden (i.e., clinicians do not need to identify and refer patients) and reduce patient burden (i.e., patients do not need to request a referral). 

The methods used to identify eligible patients for tobacco treatment have important implications, with preliminary research indicating that opt-out approaches result in the identification of a larger number of patients, higher reach and engagement [19], and higher quit rates [20], compared to opt-in approaches. Furthermore, an opt-out approach does not preclude clinician referrals.

#### 1.1.2. Centralized Delivery of Tobacco Treatment

The centralized delivery of tobacco treatment can involve the provision of services across multiple clinic and hospital locations and via multiple treatment modalities, including counseling delivered in-person or via phone or video telehealth, and the use of electronic resources (e.g., texting programs) [17]. Centralized treatment delivery, particularly by phone and video telehealth, may require fewer staff members while also providing treatment across multiple sites. Furthermore, offering multiple treatment modalities may appeal to patients’ personal preferences while also having similar abstinence rates [21,22]. Although telehealth visits have been shown to have lower no-show rates [23] and similar patient satisfaction levels [24] compared to in-person visits, disparities in digital access continue to exist [25,26], highlighting the need to offer both (non-video) phone and in-person counseling [27].

#### 1.1.3. Staff Training and Audit/Feedback

Ongoing quality improvement (QI) procedures are integral to maintaining evidence-based tobacco assessment and treatment [28,29]. While individual training is important, team training interventions can improve clinical care teamwork (e.g., communication and coordination), as well as patient mortality and morbidity [28]. Within settings of team collaboration, particularly when providing feedback, it is important to ensure a setting in which interpersonal risk-taking is supported, in order to promote communication and positive teamwork outcomes [30]. Another example of a QI method is ‘audit and feedback’, a practice used to assess performance on an individual or clinical level and to then relay this information back to the individual or practice [31,32,33]. Providing regular feedback and training improves patient care as well as collaboration among team members [28].

We utilized these three implementation strategies to achieve patient identification, outreach and enrollment, and centralized tobacco treatment and follow-up. To better understand the program’s strengths and areas in need of improvement, we conducted a QI assessment of the strategies used for program development and maintenance. Furthermore, we present the demographic, clinical, and tobacco-related characteristics of eligible patients, and the predictors of patient enrollment, treatment engagement, and abstinence in the STAR program.

## 2. Materials and Methods

### 2.1. Overview of Program Development

As recommended by the C3i leadership [7], we developed a population-based registry within specified clinics to offer tobacco treatment to all patients who currently smoke. Prior to the development of the STAR program, there had not been an oncology-based tobacco treatment program at MedStar Health. The Cancer Center leadership, oncology clinical and administrative staff, the STAR program lead, and an expert consultant (Dr. Graham Warren) identified the relevant team members and clinical champions. 

The teams made four central decisions necessary for program implementation: (1) Patient identification: To maximize the identification of patients who currently smoke, we selected an opt-out method rather than a referral-based method. One approach is for medical assistants (MAs) to document tobacco use at every outpatient visit while collecting other vital signs. This method provides more complete data collection, including showing the change in tobacco status over time, particularly important when patients were unreachable for 6-month outcome assessments. (2) Clinic location: We considered providing point-of-care tobacco treatment in conjunction with an oncology visit vs. providing separate appointments in the STAR clinic. In consultation with the clinical champions (thoracic oncologists) and clinical administrators, we determined that the busy oncology clinics and lack of available consultation rooms would not allow for point-of-care tobacco treatment. (3) Staffing of TTP: As the oncology clinicians and nursing staff were already overcommitted with oncology-related care, we determined that adding tobacco treatment to their duties would be unlikely to result in consistent, high-quality treatment. As an alternative, we elected to provide centralized treatment using multiple modalities, delivered by a full-time tobacco treatment specialist (TTS) and a part-time nurse practitioner (NP) with tobacco treatment training [34,35]. (4) EHR documentation: We determined that locating smoking status in the EHR vital signs section [36] and using a standardized single item [37] would assist with MA workflow and consistent documentation. In March 2021, MedStar’s EHR changed from Aria to Cerner, which initially reduced tobacco assessments due to the new MA workflow, but ultimately resulted in improved assessments and EHR-based communication with clinicians. 

### 2.2. Implementation Strategies and Teams

Table 1 details the relevant teams, facilitators and barriers of implementation, and the outcomes of the three implementation strategies. The TTSs were the only team members who were hired to implement the STAR program. The hematology/oncology clinical staff (e.g., MAs, NPs) and clinical administrators were all part of the existing cancer center staff with other duties. The data manager received a small percent for their effort in obtaining the numbers needed for audit and feedback. The procedures were identical across locations (MGUH and MWHC) and are described below (Table 1). Of note, although we have described the program procedures and outcomes in the past tense, the STAR program is ongoing. 

The three implementation strategies were as follows: (1) The opt-out strategy required the involvement of IT, oncology, clinical, and STAR teams. Each week, the STAR team downloaded the patients newly identified by the MAs as having smoked in <30 days [36,37]. STAR outreach staff called patients (≤3 attempts) to describe the program, enroll them and conduct a brief baseline assessment, and schedule an initial counseling session. Patients uncertain about enrolling were offered another outreach call in two months. (2) The centralized treatment strategy required actions by the STAR, IT, clinical, and pharmacy teams. The STAR staff provided treatment to both MGUH and MWHC patients (see patient-level procedures below). (3) The audit/feedback/staff training strategy required actions by the STAR, oncology administration, and clinical teams. Each month, the STAR data manager assessed the number of unique patients seen in the hematology/oncology clinics, the percentage of the patients assessed for tobacco use, and the number of patients identified as currently smoking. Using audit and feedback, we contacted the nurse manager when the percentage of patients assessed declined, who then (re)trained the MAs. Additionally, each year the STAR staff held 1–2 in-person or Zoom training sessions. The TTSs received monthly supervision from an experienced clinical psychologist. The clinicians received (1) monthly EHR updates about the status of their patients identified as currently smoking, and (2) an overview of all program metrics at the annual faculty presentation.

### 2.3. The STAR Clinical Program

Tobacco treatment specialists (TTS; 1.2 FTE) and nurse practitioners (NP; 0.2 FTE) provided up to four 30 min sessions (plus the offer of two booster sessions at the six-month assessment) of evidence-based behavioral counseling and medication, as needed. Readiness to quit was not an eligibility criterion. All patients were encouraged to engage in treatment, including those not ready to quit, and the treatment was personalized to patients’ goals. We offered first-line treatments of behavioral counseling, NRT, varenicline, and bupropion [5].

The TTS began by conducting a complete smoking history assessment, patients’ experiences with prior cessation methods, their current smoking patterns, smoking-related goals (i.e., timeframe for quitting or cutting down), and interest in medication use (NRT and/or prescription medications). From this assessment, an individualized treatment plan was developed in collaboration with the patient. Standard behavioral strategies were utilized (e.g., use of cigarette tracking, methods for reducing cigarettes per day, practice quit dates). Motivational interviewing was used to guide conversations on behavioral strategies and medication management. Quit dates were set by patients, when (and if) they became ready to make a quit attempt. For patients who were willing to consider medication use, initial and follow-up appointments were scheduled with the STAR nurse practitioner, who was also trained as a TTS. Following telehealth visits, hospital pharmacies delivered medications to patients’ homes. Due to an in-kind donation from CVS Health, since 2020 we have provided free nicotine patches and lozenges, as appropriate. Usual co-pays were required for prescription medications.

#### 2.3.1. Patient-Level Procedures

**Figure 1:**
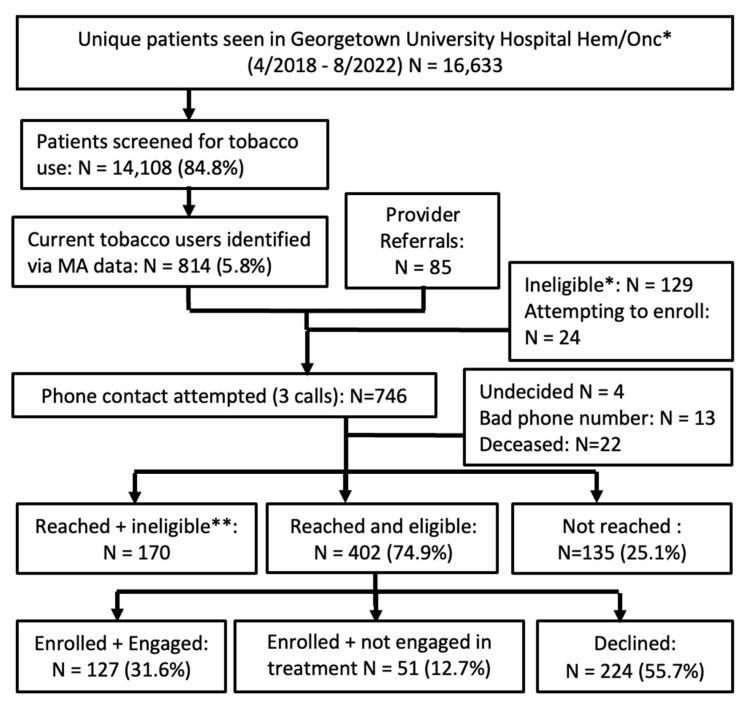
Georgetown University Hospital workflow and recruitment. * Excluding patients who did not have a visit involving a medical assistant (3/2021–8/2022). ** Reasons for ineligibility include too sick to participate as assessed via clinician, has quit/not interested in relapse prevention, no cancer/hematology dx, never smoker, second opinion, identified outside of 2 month window, hospice consultation/in hospice, no longer an MGUH patient, not fluent in English, cognitive or hearing impairment, marijuana-only smoker, vaping only. Note: as of 2022, patients who vape or are not fluent in English are now eligible for STAR.

**Figure 2:**
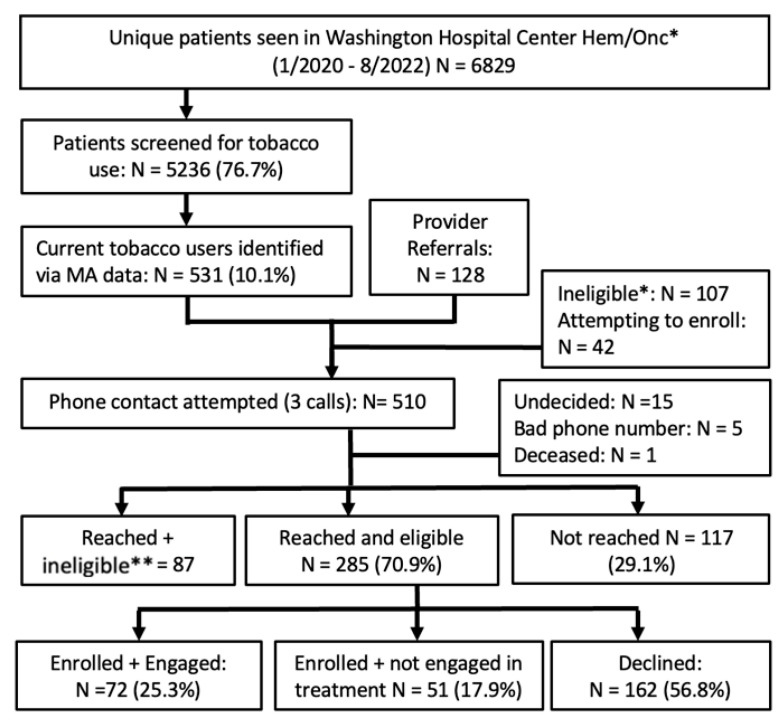
Washington Hospital Center workflow and recruitment. * Excluding patients who did not have a visit involving a medical assistant (3/2021–8/2022). ** Reasons for ineligibility include: too sick to participate as assessed via clinician, has quit/not interested in relapse prevention, no cancer/hematology dx, never smoker, second opinion, hospice consultation/in hospice, no longer an MWHC patient, not fluent in English, cognitive or hearing impairment, marijuana-only smoker, vaping only. Note: as of 2022, patients who vape or are not fluent in English are now eligible for STAR.

Using a population-based approach, the denominator includes all hematology/oncology outpatients who had an appointment within the given time period (Figure 1 and Figure 2). Using the implementation strategies described above, MAs screened patients at each outpatient visit for tobacco use. The STAR team contacted patients who had smoked in <30 days by phone, mail, and/or EHR portal for program enrollment. All assessments were completed by phone and tracked using the secure REDCap database. We contacted patients six months post-enrollment to assess current smoking status, to offer two booster sessions to patients who continued to smoke, and to solicit program feedback. 

#### 2.3.2. Patient-Level Measures

At the baseline assessment, we assessed demographic, clinical, and tobacco-related characteristics. Variables included race, ethnicity, age, sex, insurance status, primary diagnosis, and cancer stage (when applicable). Tobacco-related characteristics included smoking history (pack-years, prior quit attempts, and usage of medication and other cessation strategies). We evaluated nicotine dependence using the 2-item Heaviness of Smoking Index [38] (time to first cigarette after waking and cigarettes smoked per day), and assessed readiness to quit on a 10-point scale [39] (1 = not ready to quit, 10 = already quit). We assessed self-rated distress using a 10-point scale [40] (1 = low distress, 10 = high) wherein individuals who rated distress as a 7 or higher were referred to the psychosocial oncology team. We also ascertained whether the patient lived with others who smoked.

At the six-month assessment of MGUH patients, we evaluated 7-day point prevalence self-reported abstinence, treatment engagement (number of sessions completed and whether medications were prescribed), and program satisfaction (counseling sessions and medication offered). For patients who were unreachable at six months, we used the most recent smoking status from the MA assessments, and patients with no available data (N = 22) were classified as currently smoking. 

#### 2.3.3. Patient-Level Data Analyses

We conducted the analyses separately by site due to the differences in demographics, tobacco use, and clinical characteristics. As the MWHC program began two years after the MGUH program, we had fewer completed six-month follow-up assessments and thus predictors of abstinence were not assessed at MWHC. We used descriptive statistics and bivariate analyses (*t*-tests and X^2^ tests) to describe the associations between baseline characteristics and program enrollment (i.e., completed baseline assessment), treatment engagement (i.e., completion of ≥1 counseling session), and abstinence at MGUH (i.e., 7-day self-reported abstinence, 6 months post-enrollment). We conducted adjusted logistic regression analyses to assess predictors of engagement and abstinence at MGUH and enrollment at both sites. All regression analyses controlled for baseline demographic and clinical characteristics associated with the outcome (*p* < 0.10). We used SPSS v.27 to conduct the analyses.

## 3. Results

### 3.1. Implementation Results

Employed together, three strategies (Table 1) provided the necessary structure for program implementation, maintenance, and quality improvement. At both sites, compared to clinician referrals, the opt-out procedures resulted in the identification of the vast majority of patients who had smoked in <30 days. Over the 4.5 years of the MGUH program, 14,108/16,633 (84.8%) patients were assessed by MAs for tobacco use and 814 (5.8%) were identified as currently smoking (Figure 1). Over the 2.5 years of the MWHC program, 5236/6829 (76.7%) patients were assessed by MAs and 531 (10.1%) were identified as currently smoking (Figure 2).

The centralized treatment strategy resulted in an economy of scale as the TTSs and NPs provided evidence-based behavioral counseling and medication to patients across the two sites, through the use of phone, telehealth, and in-person visits. Due to late cancellations and no shows, phone sessions required less administrative time for TTSs than telehealth or in-person visits.

**Figure 3:**
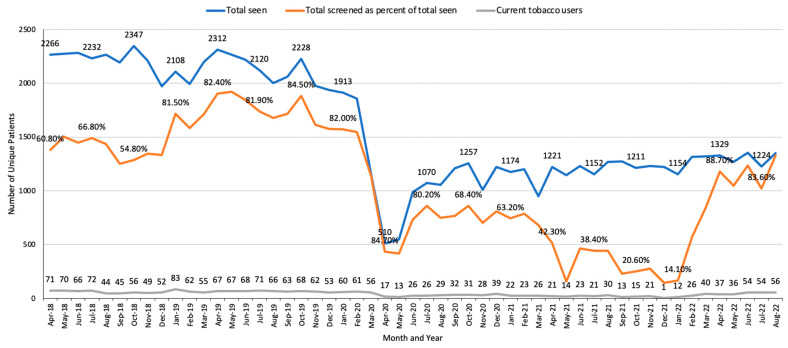
MGUH monthly assessment of the number of unique patients seen in hematology/oncology clinics, percentage of patients screened for tobacco use, and the number of identified tobacco users. Note: patients attending telehealth and lab visits were excluded from this analysis, as MAs were not involved in these visits.

**Figure 4:**
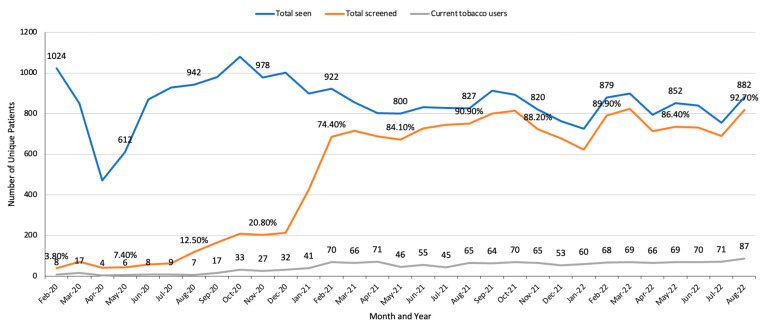
MWHC monthly assessment of the number of unique patients seen in hematology/oncology clinics, percentage of patients screened for tobacco use, and the number of identified tobacco users. Note: patients attending telehealth and lab visits were excluded from this analysis, as MAs were not involved in these visits.

Using the audit/feedback/staff training strategy, we alerted the oncology clinical team to provide feedback and training as needed to the MAs, which improved the percentage of patients assessed and identified as currently using tobacco (Figure 3 and Figure 4). For example, due to the decline in the percentage of patients assessed at MGUH in fall 2021 (Figure 3), we met with the lead MA and learned that staff shortages had led to a decline in tobacco assessments. We held a training session with the MAs and the lead MA provided individual feedback to those missing a high percentage of assessments, which increased the number of patients assessed and eligible patients identified (Figure 3). 

In Year 1 (2/2020 to 1/2021) at MWHC (Figure 4), due to the onset of the pandemic, we piloted the MA assessments only among thoracic oncology patients, which explains the disparity between the total number of patients seen and the percentage assessed for tobacco use.

### 3.2. Patient-Level Results

#### 3.2.1. MedStar Georgetown University Hospital (MGUH)

Of the 746 patients we attempted to reach, 209 were ineligible or undecided, 537 were eligible, and we reached 402 (74.9%) (Figure 1). Of those, 178 (44.3%) enrolled in the program, and 127 (31.6%) engaged in ≥one counseling session (Figure 1). The treatment modalities were as follows: 53 (41.7%) included phone only, 22 (17.3%) were in-person only, 15 (11.8%) were telehealth only, and 37 (29.1%) were a combination of modalities (of which 19 (51.4%) included at least one telehealth visit). High treatment engagement (defined as completing three+ sessions) was significantly greater among those who used a combination of modalities compared to the other modalities (X^2^ = 16.6, df = 3, *p* < 0.001).

#### 3.2.2. Baseline Predictors of Enrollment 

Compared to eligible patients who did not enroll (including never reached and eligible but declined; N = 359; 66.9%), those who did enroll (N = 178; 33.1%) were significantly more likely to be female, African American (vs. white/other racial group), to have smoked a cigarette today (vs. 1–30 days ago), to have early (vs. late) stage cancer, to have been referred by a clinician (vs. identified by MAs), and to have government-supported (vs. private) insurance (Table 2).

#### 3.2.3. Baseline Predictors of Treatment Engagement 

Among the 178 enrolled at MGUH, there was significantly greater engagement among individuals with greater nicotine dependence, greater distress, and not living with someone who was smoking (Table 2). Approaching significance, patients with early stage disease (*p* < 0.10) or who were referred by their clinicians (*p* < 0.10) were more likely to engage. A median of two counseling sessions were completed and 47.2% received NRT or prescription medications. 

Table 3 presents the adjusted logistic regression model predicting engagement. Individuals with greater baseline nicotine dependence were significantly more likely to engage in treatment (*p* = 0.009). The other bivariate predictors approached significance (*p* < 0.10).

#### 3.2.4. Baseline Predictors of Abstinence at Six Months Post-Enrollment 

Among the 178 enrolled, 146 (82%) reached the 6-month assessment (the remainder were not yet due) (Table 2). Of those, 40 (27.4%) individuals self-reported 7-day point prevalence abstinence. Furthermore, among the 146 individuals who reached the six-month assessment, 41 (28.1%) had not engaged in treatment and 105 (71.9%) had engaged in treatment. The quit rates were 8/41 (19.5%) and 32/105 (30.5%), respectively. Due to the small Ns, we have not conducted significance testing, but these differences suggest that treatment engagement may be having the expected impact on cessation.

Compared to individuals who continued to smoke, the baseline assessment indicated that those who quit were significantly less likely to have used tobacco/nicotine today (vs. 1–30 days ago), further along on the readiness to quit continuum, less likely to live with someone who smoked, and smoked fewer CPD. Furthermore, those who quit completed significantly more counseling sessions. 

Table 4 presents the adjusted logistic regression model predicting 7-day abstinence at 6 months at MGUH. After adjusting for age, nicotine dependence, and living with someone who smokes, we found that those who completed two+ counseling sessions were almost three times as likely to quit smoking, compared to those who completed zero or one session (OR = 2.80; 95% CI 1.19, 6.58). 

At the 6-month assessment, we also assessed satisfaction with and engagement in counseling: 69.8% (37/53) who completed counseling were very satisfied with the treatment and 24.5% (13/53) were somewhat satisfied. Of those engaged in treatment, 40.9% (52/127) received NRT or prescription medication. The offer of two booster sessions was accepted by 69.6% of those who had not quit, and by 40% of those who had quit (for relapse prevention). 

#### 3.2.5. MedStar Washington Hospital Center (MWHC)

Of the 510 patients who we attempted to reach, 108 were ineligible or undecided, and we reached 285 (70.9%) eligible patients (Figure 2). Of those, 123 (43.2%) enrolled in the program, and 72 (25.3%) engaged in ≥one counseling session. The treatment modalities included 33 (45.8%) phone only, 2 (2.8%) in-person only, 10 (13.9%) video telehealth only, and 27 (37.5%) combination (of which 25 (92.6%) included ≥one video telehealth visit). High treatment engagement (defined as three+ sessions) was greater among those who used a combination of modalities (44%) compared to those who used phone only (30.3%). The chi square test was not valid as there were no individuals who completed three+ sessions in the other two groups.

#### 3.2.6. Baseline Predictors of Enrollment and Engagement (Table 5)

Of the 402 eligible MWHC patients, compared to the 279 (69.4%) who did not enroll, the 123 (30.6%) who enrolled were significantly more likely to be female, to have smoked today (vs. 1–30 days ago), and to have been referred (vs. identified by MAs).

**Table 5:** MWHC baseline demographic, clinical, and tobacco-related characteristics stratified by enrollment and engagement (N = 402).

		Not Enrolled (n = 279)	Enrolled (n = 123)	Enrolled/Not Engaged ^1^ (n = 51)	Engaged (n = 72)
**Demographic Characteristics**					
**Age**	Mean (SD)	60.73 (12.1)	60.28 (9.8)	59.59 (10.6)	60.76 (9.2)
≤60	N (%)	112 (40.1) *	**61 (49.6)**	26 (51.0)	35 (48.6)
≥61	N (%)	167 (59.9)	62 (50.4)	25 (49.0)	37 (51.4)
**Sex**: Female	N (%)	155 (55.6) ***	**92 (74.8)**	38 (74.5)	54 (75.0)
**Race**					
African American or Black	N (%)	244 (88.4)	116 (94.3)	48 (94.1)	68 (94.4)
White	N (%)	28 (10.1)	5 (4.1)	2 (3.9)	3 (4.2)
Other (Asian, NHPI, Multirace, Other)	N (%)	4 (1.4)	2 (1.6)	1 (2.0)	1 (1.4)
Missing	N	3 (-)	0 (-)	0 (-)	0 (-)
**Race**					
African American or Black	N (%)	244 (87.5) *	**116 (94.3)**	48 (94.1)	68 (94.4)
White or other (Asian, NHPI ^2^, Multirace, Other)	N (%)	32 (11.5)	7 (5.7)	3 (5.9)	4 (5.6)
Missing	N	4 (-)	0 (-)	0 (-)	0 (-)
**Ethnicity**					
Hispanic or Latino	N (%)	4 (1.5)	1 (0.8)	0 (0.0)	1 (1.4)
Non-Hispanic		268 (98.5)	120 (99.2)	49 (100.0)	71 (98.6)
Missing/unknown		7 (-)	2 (-)	2 (-)	0 (-)
**Insurance Type**					
Private	N (%)	69 (24.7)	29 (23.6)	7 (13.7)	22 (30.6)
Medicare or military	N (%)	136 (48.7)	50 (40.7)	23 (45.1)	27 (37.5)
Medicaid or other government plan	N (%)	74 (26.5)	44 (35.8)	21 (41.2)	23 (31.9)
**Clinical Characteristics**					
**Tobacco-Related Cancer ^3^**					
Tobacco-related cancer	N (%)	76 (27.2)	33 (26.8)	11 (21.6)	22 (30.6)
Non-tobacco-related cancer	N (%)	139 (49.8)	61 (49.6)	26 (51.0)	35 (48.6)
N/A: hematologic or diagnostic or screening	N (%)	64 (22.9)	29 (23.6)	14 (27.5)	15 (20.8)
**Diagnosis**					
Cancer stage 0, I, II	N (%)	88 (34.8) *	**54 (46.6)**	24 (50.0)	30 (44.1)
Cancer stage III or IV	N (%)	103 (40.7)	35 (30.2)	12 (25.0)	23 (33.8)
Non-cancer hematological diagnosis	N (%)	62 (24.5)	27 (23.3)	12 (25.0)	15 (22.1)
N/A: diagnostic or cancer screening	N	2 (-)	2 (-)	2 (-)	0 (-)
Missing/unknown	N	24 (-)	5 (-)	1 (-)	4 (-)
**Method Patient Identified**					
Clinician referral	N (%)	39 (14.0) ***	**63 (51.2)**	28 (54.9)	37 (51.4)
MA assessment	N (%)	240 (86.0)	60 (48.8)	23 (45.1)	35 (48.6)
**Psychological Distress ^4^ (1–10, high = more)**					
≤6	N (%)	--	--	28 (54.9) *	51 (70.8)
≥7	N (%)	--	--	23 (45.1)	21 (29.2)
**Tobacco-Related Characteristics**					
**Smoking Status (MA Assessment)**					
Smoked a cigarette today	N (%)	195 (73.9) **	**103 (83.7)**	45 (88.2)	58 (80.6)
Smoked 1–30 days ago	N (%)	69 (26.1)	20 (16.3)	6 (11.8)	14 (19.4)
Used other nicotine/tobacco (not cigarettes)		15 (-)	0 (-)	0 (-)	0 (-)
**Cigarettes per day (number)**	Mean (SD)	--	--	11.7 (10.2) *	**9.1 (6.1)**
	Median	--	--	10.0	7.0
**Cigarettes per day (categorical)**					
≤5	N (%)	--	--	13 (25.5)	25 (35.2)
6 to 10	N (%)	--	--	19 (37.3)	24 (33.8)
≥11	N (%)	--	--	19 (37.3)	22 (31.0)
Missing				0 (-)	1 (-)
**Pack-years**	Mean (SD)	--	--	29.8 (19.0)	37.3 (31.0)
	Median	--	--	23.0	32.4
**Time to first cigarette after waking**					
<30 min	(N, %)	--	--	34 (66.7)	46 (65.7)
31–60 min	(N, %)	--	--	8 (15.7)	12 (17.1)
61+ min	(N, %)	--	--	9 (17.6)	12 (17.1)
Missing/refused	N	--	--	0 (-)	2 (-)
**Readiness to Quit**					
Not ready to quit (≥6 months)	(N, %)	--	--	21 (41.2)	24 (34.3)
Ready to quit <30 days	(N, %)	--	--	29 (56.9)	45 (64.3)
Already quit (<30 days)	(N, %)	--	--	1 (2.0)	1 (1.4)
Missing/refused	N	--	--	0 (-)	2 (-)
**Lives With Person Who Smokes**					
No (or lives alone)	(N, %)	--	--	34 (66.7)	51 (70.8)
Yes	(N, %)	--	--	17 (33.3)	21 (29.2)
**Treatment Engagement**					
**Sessions Completed (Number)**	Mean (SD)	--	--	n/a	2.04 (1.1)
	Median			n/a	2.0
**Sessions Completed (Categorical)**					
0/1 counseling sessions	N (%)	--	--	n/a	30 (41.7)
2+ sessions	N (%)	--	--	n/a	42 (58.3)
**STAR Prescription or NRT**					
Yes	N (%)	--	--	n/a	43 (59.7)

Notes. * *p* = 0.10, ** *p* < 0.05, *** *p* < 0.001. ^1^ Enrolled/not engaged are those who completed the baseline assessment but did not engage in counseling. ^2^ NHPI: Native Hawaiian and Pacific Islander; MA: medical assistant; ^3^ tobacco-related cancers included lung, head, and neck, stomach, kidney, pancreas, liver, bladder, cervix, colorectal, and acute myeloid leukemia; ^4^ patients reporting a score of 7 to 10 were offered a referral to the Psychosocial Oncology Program for evaluation and treatment. Bolded text indicates significant findings.

Among those who enrolled (N = 123), there were no significant demographic, clinical, or tobacco-related differences between those who engaged (N = 72; 58.5%) vs. those who did not engage (N = 51; 41.5%) in treatment. As there were only two nonsignificant trends, we did not conduct a logistic regression analysis. Regarding engagement, patients completed a median of two counseling sessions and 59% received NRT or prescription medication.

Finally, of the 123 enrolled, only 81 (65.9%) had reached the six-month assessment point, and of those, 12 (14.8%) self-reported 7-day point-prevalence abstinence. Due to the limited numbers available at present, we did not assess predictors of abstinence at MWHC. Furthermore, among the 81 individuals who had reached the six-month assessment, 29 (35.8%) had not engaged in treatment and 52 (64.2%) had engaged in treatment. The quit rates were 2/29 (6.9%) and 10/52 (19.2%), respectively. As above, these Ns are too small to conduct significance testing, but are in the expected direction.

#### 3.2.7. Predictors of Enrollment at MGUH and MWHC

Table 6 presents the adjusted logistic regression models predicting enrollment. At MGUH, compared to those who declined enrollment, those who enrolled were significantly more likely to be African American (vs. white/other) and referred to STAR (vs. identified by MAs). At MWHC, there were similar results, with those referred more likely to enroll, and trends for African Americans and older adults in terms of being more likely to enroll (vs. white/other races and younger adults, respectively). 

## 4. Discussion

Tobacco use among patients with cancer results in poorer treatment outcomes, increased mortality, and treatment-related toxicities [3]. However, as the necessary resources and expertise are often unavailable in the oncology setting, patients frequently do not receive tobacco use treatment to stop smoking. As part of the NCI’s C3i initiative and in collaboration with MedStar Health, we implemented, expanded, and have sustained the STAR program in two hematology/oncology settings. This has required the involvement of multiple oncology-based teams, including cancer center leadership, IT programmers and informaticists, clinical administrators, clinical staff, pharmacists, and the tobacco treatment team. The teams’ utilization of implementation strategies to identify, reach, and enroll patients in an evidence-based tobacco treatment program has resulted in program expansion and maintenance.

To assess the program’s strengths and limitations, we conducted a QI assessment of the implementation strategies and an analysis of the patient-level predictors of enrollment, treatment engagement, and abstinence. A total of 19,344 unique patients were assessed for tobacco use over two clinical sites, which averaged to a 7% rate of current tobacco use, comparable to other C3i sites [41] and to national rates of smoking among older patients with cancer [42]. The difference in smoking rates by site is unclear, but may be explained in part by the small sample size at MWHC and the higher percentage of individuals with private insurance at MGUH [43].

By leveraging key implementation strategies, including the opt-out approach and audit/feedback with the MAs, we sought to address inequities in cessation treatment, which resulted in reaching and engaging a majority-African-American patient population [19,44]. This is important, as African Americans and other minoritized groups are less likely to receive assistance to quit smoking from their clinicians [44,45]. The QI results revealed the importance of both the opt-out approach for tobacco assessment and the inclusion of clinician referrals. The opt-out approach provided a population-based registry of tobacco use across the specified clinics, the identification of more patients than is feasible in a referral-based program, and an assessment of the program’s reach, treatment engagement, and abstinence outcomes. Involving clinicians in the referral process and regularly informing them of their patients’ outcomes gave clinicians the information needed to engage with their patients about tobacco use. Importantly, patients referred by their clinicians were more likely to enroll in the program at both sites, and marginally more likely to engage in treatment at the MGUH site. Based on these results showing significantly higher enrollment following a clinician referral, we plan to modify the EHR so that clinicians are prompted to refer their currently smoking patients to STAR.

The percentage of patients who engaged in treatment was 31.6% at MGUH (Figure 1) and 25.3% at MWHC (Figure 2), both higher than the 18.4% median across 28 C3i programs [41]. Similarly, the 6-month abstinence rate at MGUH was 27.4% higher than the 18.4% median of the C3i programs, and with data collected on 85% of those enrolled. The abstinence rate at MWHC was lower at 14.8%, which we attribute in part to the smaller sample that had reached the 6-month assessment and thus requires continued assessment as the MWHC matures. Although there is certainly room for improvement, we attribute these successes to the teams-based approach and the implementation strategies utilized to implement, expand, and maintain the program.

The multivariate analyses predicting engagement at MGUH indicated that patients with greater nicotine addiction at baseline were significantly more likely to engage in treatment. This finding suggests the importance of offering pharmacotherapy to all patients engaged in treatment, particularly those with greater addiction. Our data indicated that we can improve in this area, as an average of one half of the engaged patients received medication from the program, possibly suggesting that not all patients understood the benefits of medication and/or its availability (i.e., free NRT or prescription medication that is covered by insurance). Furthermore, improved communication regarding the importance of engaging in behavioral counseling may be needed for patients smoking a few cigarettes a day, as their lower engagement may suggest less motivation to stop smoking and/or have a plan to quit on their own [41]. Efforts to increase patient engagement may benefit from communicating our findings to patients who are uncertain about engaging in treatment, such that patients who completed two+ counseling sessions were almost three times as likely to report abstinence compared to those who completed ≤1 session. 

Based on the implementation and success of the STAR program, there are two new programs at MedStar Health designed to assess and treat tobacco use. First, with funding from the DC Department of Health, we are expanding tobacco assessment (conducted by MAs) to primary care departments, which will prompt clinicians to consider tobacco treatment, including medication prescriptions and/or e-referral to the tobacco quitline. Clinicians are also prompted to consider referral for lung cancer screening among potentially eligible patients. Second, as part of the Commission on Cancer’s ‘Just Ask’ Program [46], MedStar and Lombardi administrators have employed the same single item opt-out tobacco assessment [37] in all oncology clinics across MedStar Health. As above, clinicians are prompted to consider cessation medications and/or e-referral to the quitline. Additional funding will be required in order for the STAR program to expand and provide tobacco use treatment for oncology patients across MedStar Health. 

The program’s limitations include the following. First, the data associated with the clinical program are not fully integrated into the EHR. Due to the time and expense required for making additional EHR modifications at the program’s outset, we elected to record patient responses in a parallel secure database (REDCap). Although the MAs’ tobacco assessment is accessible in the EHR, it is not widely utilized by clinicians, and thus requires an EHR modification to increase STAR referrals and clinician-initiated discussions about cessation. Second, despite regular audit, feedback, and training of staff, we have only recently approached 80% completion of tobacco assessment for all patients. This finding indicates that program maintenance requires continuous audit, feedback, and training, regardless of the amount of time since program outset. Finally, we have not yet conducted an economic analysis of the program and will undertake this effort once both sites have reached a steady state. In a recent paper, Salloum and colleagues [47] described an economic analysis of 15 of the C3i programs and concluded that they were “within the range of historical cost-effectiveness estimates of tobacco treatment…”.

## 5. Conclusions

In summary, the systematic utilization of multiple oncology-based teams resulted in the implementation, expansion, and maintenance of the STAR program. From this QI assessment and presentation of patient-level results, we have demonstrated the program’s successes in engagement and cessation rates, as well as areas in need of improvement. For example, although the opt-out method was central for identifying patients who were using tobacco, bolstering referrals from all oncology clinicians is needed to expand treatment engagement among patients. Oncology clinics and departments planning to develop a tobacco use treatment program may benefit from our lessons learned and from employing the teams and implementation strategies described here.

## Figures and Tables

**Table 1:** Strategies and teams for program implementation and maintenance.

Strategy	Teams Involved	Team Actions	Facilitators	Barriers	Outcomes
**Opt-out approach** to identify and reach all new patients who have smoked tobacco in the past 30 days	EHR IT team (programmer, provider informaticist) and clinical administration team (Dir. of Oncology Services)	EHR modifications of MA workflow and development of weekly reporting tools.	Clinical administration team helps to obtain modifications prioritized by the EHR team.	Low priority of work requests submitted to EHR programmers results in delays in improvements.	Number (%) of all hem/onc patients assessed for tobacco use (monthly and overall). (See Figure 1, Figure 2, Figure 3 and Figure 4.)
	Clinical team: medical assistants (MAs) and Dir. of Nursing Services	MAs document tobacco status at every visit and provide a STAR flyer to those who have used tobacco or nicotine in <30 days.	Monthly audit and feedback with lead MAs on percentage of patients assessed for tobacco use.	MAs’ time constraints, need for regular training, and staff turnover.	Identification of more current smokers compared to referral methods (see Figure 1 and Figure 2).
	STAR team: TTS, NP, outreach staff (interns), data manager, clinical psychologist supervisor for TTS	Outreach and intake completion of new patients (smoked in < 30 days) via phone, email, and/or EHR portal.	Availability of staff time; shared Google voice line allows staff to return calls from office or home; monthly EHR messages sent to providers re status of their patients.	Limited integration of STAR procedures with EHR (e.g., the contact attempts made to STAR patients are not tracked in EHR).	Tracking data on % reached and % enrolled (see Figure 1 and Figure 2).
**Centralized approach** to providing tobacco treatment at 2 hospitals	STAR team	TTS phone-based sessions: TTS provided evidence-based behavioral counseling and assessed potential need for medication and NP visit.	Use of Google calendar for immediate scheduling of counseling during the intake call; phone-based calls reduce staff time needed.	Patient cancellations/no shows/rescheduling requires staff time.	Tracking data on number of counseling sessions completed; phone sessions require less time for TTSs and patients than telehealth or in-person visits.
	Administrative, STAR, clinical, and pharmacy teams	In-person visits: NPs and TTSs collaborate on medication and behavioral strategies. Pharmacy stores and distributes NRT donated by CVS.	Admin team identified NPs with available time for STAR and needed the clinical space; NP and TTS review prior session(s) in advance of the upcoming session.	Clinical space for the visits; limited clinic availability reduces options for patients.	TTSs provide treatment to patients at two different hospitals, providing greater efficiency.
	STAR, clinical, IT, and pharmacy teams	Telehealth visits: The IT team set up telehealth procedures after pandemic onset. NPs and TTSs provide same counseling and medication options as for in-person visits.	When available, technical assistance is provided to connect patient and NPs for the sessions; otherwise, regular phone calls were used when telehealth calls failed or were not possible.	State licenses necessary for MD and VA; some patients have connection issues with telehealth platform.	Patients receive counseling at home. Medications are delivered to patients’ homes.
**Audit/feedback/training**	STAR and clinical teams	STAR data manager and staff audit MA tobacco assessments each month.	Feedback provided to lead MA who communicates with MA team; STAR team meets with clinical team every 6 months to review procedures.	High MA turnover, busy clinics.	Data provide % of patients who are assessed for tobacco use each month. Feedback and training result in higher % of patients assessed; (see Figure 3 and Figure 4).
	STAR, oncology admin, and clinical teams	Attendance at STAR meetings every other month.	Regular attendance by all team members; remote meetings assist with attendance.	Lack of attendance due to competing priorities and staff shortages.	Gain input from multiple teams and address issues more quickly than via email exchanges.
	STAR team	Monthly notifications sent to providers via EHR on their patients’ enrollment and smoking status.	Buy-in and awareness of providers improves when updated on their patients’ progress; providers appreciate receiving the updates on their patients.	Limited time for providers to review messages and encourage patients regarding quitting and remaining smoke-free.	Providers are kept informed and can reinforce cessation at visits; messages remind providers to refer other patients.
	STAR team	TTS meets monthly with clinical psychologist supervisor.	Clinical psychologist supervises TTSs on motivational interviewing and behavioral cessation strategies.	Limited time to discuss all patients.	TTS uses feedback to improve counseling for patients.
	Clinical admin and STAR teams	Attend standing faculty meeting for annual STAR updates and review of data on outreach, treatment engagement, and abstinence.	Updates provided during regularly scheduled faculty meeting. Provider permission to contact patients without a referral is confirmed.	Providers’ time and interest; time available on meeting agenda is limited.	Providers are reminded of the rationale for tobacco treatment, institutional program support, and to refer their patients.

**Table 2:** MGUH baseline demographic, clinical, and tobacco-related characteristics, by enrollment, engagement, and abstinence.

		Not Enrolled (n = 359)	Enrolled (n = 178)	Enrolled/Not Engaged ^1^ (n = 51)	Engaged (n = 127)	6-Month Not Quit (n = 106)	6-Month Quit (n = 40)
**Demographics**							
**Age**	M (SD)	59.8 (10.6)	59.8 (12.6)	59.3 (10.2)	59.9 (10.8)	59.2 (10.9)	60.8 (11.2)
≤60	N (%)	158 (44.0)	84 (47.2)	27 (52.9)	57 (44.9)	54 (50.9) *	**14 (35.0)**
≥61	N (%)	201 (56.0)	94 (52.8)	24 (47.2)	70 (55.1)	52 (49.1)	26 (65.0)
**Sex**							
Female	N (%)	165 (46.0) ***	**105 (59.0)**	29 (56.9)	76 (59.8)	66 (62.3)	22 (55.0)
**Race**							
African American	N (%)	148 (42.7) ***	**113 (64.9)**	35 (70.0)	78 (62.9)	64 (62.1)	27 (67.5)
White	N (%)	166 (47.8)	46 (26.4)	14 (28.0)	32 (25.8)	31 (30.1)	8 (20.0)
Other (e.g., Asian, NHPI ^2^)	N (%)	33 (9.5)	15 (8.6)	1 (2.0)	14 (11.3)	8 (7.8)	5 (12.5)
Missing		12 (-)	4 (-)	1 (-)	3 (-)	3 (-)	0 (-)
**Ethnicity**							
Hispanic or Latino	N (%)	15 (4.4)	9 (5.3)	1 (2.0)	8 (6.6)	4 (4.0)	3 (7.5)
Non-Hispanic	N (%)	328 (95.6)	162 (94.7)	49 (98.0)	113 (93.4)	97 (96.0)	37 (92.5)
Missing/unknown	N (%)	16 (-)	7 (-)	1 (-)	6 (-)	5 (-)	0 (-)
**Insurance Type**							
Private	N (%)	170 (47.6) **	**64 (36.2)**	20 (39.2)	44 (34.9)	39 (36.8)	16 (40.0)
Medicare or military	N (%)	145 (40.6)	86 (48.6)	22 (43.1)	64 (50.8)	50 (47.2)	19 (47.5)
Medicaid or other govt	N (%)	42 (11.8)	27 (15.3)	9 (17.6)	18 (14.3)	17 (16.0)	5 (12.5)
No insurance/unknown	N (%)	1 (-)	1 (-)	0 (-)	1 (-)	0 (-)	0 (-)
**Clinical Variables**							
**Primary Diagnosis**							
Breast cancer	N (%)	43 (12.0)	24 (13.5)	4 (7.8)	20 (15.7)	15 (14.2)	6 (15.0)
Head/neck cancer	N (%)	14 (3.9)	4 (2.2)	0 (0.0)	4 (3.1)	2 (1.9)	1 (2.5)
Gastrointestinal cancer	N (%)	73 (20.3)	27 (15.2)	10 (19.6)	17 (13.4)	14 (13.2)	2 (5.0)
Genitourinary cancer	N (%)	52 (14.5)	11 (6.2)	4 (7.8)	7 (5.5)	3 (2.8)	5 (12.5)
Leukemia/lymphoma	N (%)	25 (7.0)	15 (8.4)	3 (5.9)	12 (9.4)	12 (11.3)	3 (7.5)
Lung cancer	N (%)	42 (11.7)	38 (21.3)	12 (23.5)	26 (20.5)	22 (20.8)	10 (25.0)
Other (brain, gyn, skin)	N (%)	51 (14.2)	23 (12.9)	9 (17.6)	15 (11.8)	12 (11.3)	6 (15.0)
Heme Dx (e.g., anemia, DVT)	N (%)	44 (12.3)	21 (11.8)	6 (11.8)	15 (11.8)	14 (13.2)	6 (15.0)
Lung cancer screening	N (%)	6 (1.7)	13 (7.3)	3 (5.9)	10 (7.9)	11 (10.4)	0 (0.0)
Diagnostic, other, non-LCS	N (%)	9 (2.5)	2 (1.2)	0 (0.0)	2 (1.6)	1 (0.9)	1 (2.5)
**Tobacco-Related Cancer ^3^**							
Yes	N (%)	156 (43.5)	75 (42.1)	21 (41.2)	54 (42.5)	39 (36.8)	17 (42.5)
No	N (%)	144 (40.1)	68 (38.2)	22 (43.1)	46 (36.2)	42 (39.6)	16 (40.0)
N/A: heme or diagnostic	N (%)	59 (16.4)	35 (19.7)	8 (15.7)	27 (21.3)	25 (23.6)	7 (17.5)
**Cancer Stage**							
Cancer stage 0, I, II	N (%)	94 (29.4) *	**61 (39.9)**	14 (29.2) *	**47 (44.8)**	36 (41.4)	17 (45.9)
Cancer stage III or IV	N (%)	180 (56.3)	70 (45.8)	28 (58.3)	42 (40.0)	36 (41.4)	14 (37.8)
N/A: non-cancer hem dx	N (%)	46 (14.4)	22 (14.4)	6 (12.5)	16 (15.2)	15 (17.2)	6 (16.2)
N/A: diagnostic, ca screen	N	13 (-)	13 (-)	3 (-)	10 (-)	11 (-)	1 (-)
Missing/unknown	N	26 (-)	26 (-)	0 (-)	12 (-)	8 (-)	2 (-)
**Method Patient Identified**							
Clinician referral	N (%)	24 (6.7) ***	**60 (33.7)**	12 (23.5) *	**48 (37.8)**	39 (36.8)	15 (37.5)
MA assessment	N (%)	335 (93.3)	118 (66.3)	39 (76.5)	79 (62.2)	67 (63.2)	25 (62.5)
**Psych. Distress ^4^** (1 = low to 10 = high)		--	--				
≤6	M (SD)	--	--	26 (60.5) **	**49 (41.5)**	17 (47.2)	42 (44.7)
≥7	M (SD)	--	--	17 (39.5)	69 (58.5)	19 (52.8)	52 (55.3)
Missing	N	--	--	8 (-)	9 (-)	4 (-)	12 (-)
**Baseline Tobacco Variables**							
**MA Smoking Assessment**							
Smoked a cigarette today	N (%)	233 (69.3) *	**137 (77.4)**	38 (74.5)	99 (78.6)	**86 (81.9)**	24 (60.0) ***
Smoked 1–30 days ago	N (%)	103 (30.7)	40 (22.6)	13 (25.5)	27 (21.4)	19 (18.1)	16 (40.0)
Used other nicotine/tobacco		23 (-)	1 (-)	0 (-)	1 (-)	1 (-)	0 (-)
**Cigarettes Per Day** (N)	M (SD)	--	--	9.1 (7.8)	10.6 (9.5)	11.4 (9.2) **	**7.6 (8.3)**
	Median	--	--	6.5	9.0	10.0	6.0
**Cigarettes Per Day** (categ.)							
≤5	N (%)	--	--	20 (40.0)	47 (37.3)	31 (29.8) *	**19 (47.5)**
6 to 10	N (%)	--	--	18 (36.0)	41 (32.5)	38 (36.5)	14 (35.0)
≥11	N (%)	--	--	12 (24.0)	38 (22.4)	35 (33.7)	7 (17.5)
**Pack-Years**	M (SD)	--	--	31.9 (25.0)	36.6 (28.0)	34.1 (26.1)	34.7 (31.5)
	Median	--	--	22.5	29.5	27.0	24.25
**First Cigarette After Waking**		--	--				
<30 min	(N, %)	--	--	23 (45.1) *	**73 (63.5)**	58 (59.2)	18 (47.4)
31–60 min	(N, %)	--	--	12 (23.5)	18 (15.7)	18 (18.4)	5 (13.2)
61+ min	(N, %)	--	--	16 (31.4)	24 (20.9)	22 (22.4)	15 (39.5)
Missing/refused	N	--	--	0 (-)	12 (-)	8 (-)	2 (-)
**Readiness to Quit**		--	--				
Not ready to quit (≥6 mos)	(N, %)	--	--	18 (35.3)	38 (34.2)	39 (41.1) ***	**7 (18.4)**
Ready to quit ≤30 days	(N, %)	--	--	24 (47.1)	64 (57.7)	51 (53.7)	19 (50.0)
Already quit (<30 days)	(N, %)	--	--	9 (17.6)	9 (8.1)	5 (5.3)	12 (31.6)
Missing/refused	N	--	--	0 (-)	16 (-)	11 (-)	2 (-)
**Lives w. Person Smoking**		--	--				
No (or lives alone)	(N, %)	--	--	29 (56.9) **	**90 (74.4)**	68 (66.7) **	**33 (84.6)**
Yes	(N, %)	--	--	22 (43.1)	31 (25.6)	34 (33.3)	6 (15.4)
Missing	N	--	--	0 (-)	6 (-)	4 (-)	1 (-)
**STAR Engagement**							
**Sessions Complete**	M (SD)	--	--	n/a	2.52 (1.6)	1.62 (1.7) **	**2.35 (1.8)**
	Median	--	--	n/a	2.0	1.0	3.0
**Sessions Complete** (categ.)		--	--				
0/1 sessions	N (%)	--	--	n/a	43 (33.9)	61 (57.5) *	**16 (40.0)**
2+ sessions	N (%)	--	--	n/a	84 (66.1)	45 (42.5)	24 (60.0)
**STAR Prescription or NRT**		--	--				
Yes	N (%)	--	--	n/a	60 (47.2)	38 (35.8)	13 (32.5)

Notes. * *p* = 0.10, ** *p* < 0.05, *** *p* < 0.001. ^1^ Enrolled/not engaged are those who completed the baseline assessment but did not engage in counseling; ^2^ NHPI: Native Hawaiian and Pacific Islander; MA: medical assistant; ^3^ tobacco-related cancers included lung, head, and neck, stomach, kidney, pancreas, liver, bladder, cervix, colorectal, and acute myeloid leukemia; ^4^ patients reporting a score of 7 to 10 were offered a referral to the Psychosocial Oncology Program for evaluation and treatment. Bolded text indicates significant findings.

**Table 3:** Adjusted logistic regression analysis: predicting treatment engagement at MGUH.

MGUH (n = 125)		
	OR (95% CI)	*p* Value
**Diagnosis**		
Stage 0, I, II	1	
Stage III or IV	0.46 (0.19–1.16)	0.099
Hematologic condition	0.98 (0.25–3.92)	0.976
**Method of patient identification**		
MA assessment	1	
Provider referral	2.64 (0.86–8.06)	0.088
**Time to first cigarette after waking**		
After 60 min	1	
31 to 60 min	1.53 (0.46–5.14)	0.489
Within 30 min	**3.92 (1.40–10.96)**	**0.009**
**Lives with smoker**		
No or lives alone	1	
Yes	0.42 (0.17–1.04)	0.061
**Distress score (0–10)**		
6 or lower	1	
7+	2.31 (0.97–5.48)	0.059

Note. Bolded text indicates significant findings.

**Table 4:** Adjusted logistic regression analysis: predicting 7-day point prevalence abstinence at 6 months at MGUH.

MGUH (n = 131)		
	OR (95% CI)	*p* Value
**Age groups**		
60 and under	1	
61+	1.27 (0.54–2.95)	0.583
**Time to first cigarette after waking**		
Within 30 min	1	
31 to 60 min	0.92 (0.25–3.37)	0.895
After 60 min	2.18 (0.85–5.61)	0.105
**Last day smoked**		
Today	1	
One or more days ago	2.48 (0.96–6.37)	0.060
**Lives with smoker**		
No or lives alone	1	
Yes	0.51 (0.18–1.47)	0.215
**Number of counseling sessions**		
0 to 1	1	
2+	**2.80 (1.19–6.58)**	**0.018**

Note. Bolded text indicates significant findings.

**Table 6:** Adjusted logistic regression analysis: predicting enrollment in STAR.

	Model 1: MGUH (n = 437)		Model 2: MWHC (n = 353)	
	OR (95% CI)	*p* Value	OR (95% CI)	*p* Value
**Age**				
≤60	—		1	
≥61	—		0.64 (0.38–1.06)	0.084
**Sex**				
Male	1		1	
Female	1.38 (0.89–2.13)	0.153	1.43 (0.82–2.51)	0.208
**Race**				
White or other	1		1	
Black/African American	**2.59 (1.64–4.10)**	**<0.001**	2.23 (0.87–5.69)	0.093
**Diagnosis**				
Stage 0, I, II cancer	1		1	
Stage III or IV cancer	0.80 (0.50–1.30)	0.372	0.72 (0.40–1.30)	0.280
Hematologic condition	0.89 (0.45–1.76)	0.728	0.88 (0.45–1.70)	0.697
**Insurance type**				
Private insurance	1		–	
Medicare or military plan	1.51 (0.95–2.40)	0.084	–	
Medicaid or other govt-sponsored	1.01 (0.50–2.01)	0.984	–	
**MA tobacco assessment**				
1 or more days ago	1		1	
Smoked today	1.29 (0.79–2.09)	0.315	1.44 (0.77–2.69)	0.249
**Method of patient identification**				
MA assessment	1		1	
Provider referral	**6.21 (3.25–11.89)**	**<0.001**	**5.86 (3.39–10.13)**	**<0.001**

Note. Bolded text indicates significant findings.

## Data Availability

Deidentified data presented in this quality improvement project are available upon request from the corresponding author. The data are not publicly available as this was a quality improvement analysis and not a research study.
